# Application of Rice Husk-Derived SBA-15 Bifunctionalized with C18 and Sulfonic Groups for Solid-Phase Extraction of Tropane, Pyrrolizidine, and Opium Alkaloids in Gluten-Free Bread

**DOI:** 10.3390/foods14071156

**Published:** 2025-03-26

**Authors:** Fernando L. Vera-Baquero, Judith Gañán, Natalia Casado, Damián Pérez-Quintanilla, Sonia Morante-Zarcero, Isabel Sierra

**Affiliations:** 1 Departamento de Tecnología Química y Ambiental, E.S.C.E.T, Universidad Rey Juan Carlos, C/Tulipán s/n, 28933 Móstoles, Spain; fernando.vera@urjc.es (F.L.V.-B.); judith.ganan@urjc.es (J.G.); natalia.casado@urjc.es (N.C.); damian.perez@urjc.es (D.P.-Q.); 2 Instituto de Investigación de Tecnologías para la Sostenibilidad, Universidad Rey Juan Carlos, 28933 Móstoles, Spain

**Keywords:** rice husk, bifunctionalized SBA-15, solid-phase extraction, tropane alkaloids, pyrrolizidine alkaloids, opium alkaloids, multifamily analytical methodology, food control

## Abstract

Rice husk (RH), a globally abundant agri-food waste, presents a promising renewable silicon source for producing SBA-15 mesoporous silica-based materials. This study aimed to synthesize and bifunctionalize SBA-15 using RH as a silica precursor, incorporating sulfonic and octadecyl groups to create a mixed-mode sorbent, RH-SBA-15-SO_3_H-C18, with reversed-phase and cation exchange properties. The material’s structure and properties were characterized using advanced techniques, including X-ray diffraction, infrared spectroscopy, N_2_ adsorption–desorption isotherms, nuclear magnetic resonance, and electron microscopy. These analyses confirmed an ordered mesoporous structure with a high specific surface area of 238 m^2^/g, pore volume of 0.45 cm^3^/g, pore diameter of 32 Å, and uniform pore distribution, highlighting its exceptional textural qualities. This sorbent was effectively utilized in solid-phase extraction to purify 29 alkaloids from three families—tropane, pyrrolizidine, and opium—followed by an analysis using ultra-high performance liquid chromatography coupled to ion-trap tandem mass spectrometry. The developed analytical method was validated and applied to gluten-free bread samples, revealing tropane and opium alkaloids, some at concentrations exceeding regulatory limits. These findings demonstrate that RH-derived RH-SBA-15-SO_3_H-C18 is a viable, efficient alternative to commercial sorbents for monitoring natural toxins in food, offering a sustainable solution for repurposing agri-food waste while addressing food safety challenges.

## 1. Introduction

Plant toxins of natural origin, particularly some alkaloids, such as pyrrolizidine (PAs), tropane (TAs) and opium (OAs) alkaloids, have received special attention in the recent years among the food science community. The intake of these toxins could lead to serious health problems, from acute poisoning to the development of chronic diseases or even death [1,2,3,4]. For this reason, controlling these alkaloids is currently one of the main priorities within the food safety field, as there is an exponential number of food alerts that have reported significant concentration levels in different foods [5,6]. In fact, the European regulation of these compounds in food began recently (2020 for PAs and 2021 for TAs and OAs) [7,8,9]. However, due to the increasing number of alerts and advances in research on these alkaloids, these regulations have been recently reviewed and updated, leading to Commission Regulation (EU) 2023/915, which is currently in force [10]. This regulation includes maximum concentration limits of these toxins for some food products. Moreover, an additional regulation related to the methods of sampling and analysis used to control these alkaloids in food has also been published (Commission Implementing Regulation (EU) 2023/2783) [11].

Accordingly, there is a great interest in developing analytical methodologies that enable the simultaneous control of these three types of alkaloids in food to comply with the current regulations and contribute to ensuring food safety. Nonetheless, controlling these alkaloids in food is not an easy task, since they may sometimes be present at low concentration levels (µg/kg). Likewise, their identification and quantification can be hindered by matrix interferences due to the high complexity of food samples. Also, the development of multicomponent methods entails some difficulties since suitable analytical conditions must be achieved for the different analytes. For these reasons, sample preparation is a crucial step for an accurate and simultaneous determination of these alkaloids in food samples [12,13].

The current trend in the development of analytical methods is to design green strategies that comply with the Green Chemistry principles, which are in line with some of the Sustainable Development Goals, such as SDG No. 12 (responsible consumption and production) and SDG No. 13 (climate action) [14,15]. Among these strategies, the development of green materials for sorbent-based procedures can be highlighted. For this purpose, one approach is to synthesize new nanomaterials using natural sources, such as the use of agri-food waste (e.g., fruit peels, eggshells, or cereal husks) [16] In this sense, rice husk (RH), which is known to be an excellent source of energy, widely used in methane and hydrogen production [17], can be an interesting alternative to produce functional materials due to the high siliceous composition of their ashes, which is higher than 90% on a dry basis [18]. In short, RH ash is an economical and widely available raw material suitable for obtaining silica-based materials [19], providing a possible solution to the environmental problem of disposing of the ashes after energy production, and ensuring a circular economy [20].

Mesostructured silicas (MSs) are silicate networks with a remarkably organized structure, large pore size, and high surface area. Moreover, they are very stable materials, and their surface can be easily modified with specific functional groups, which provides them with a high versatility [21]. Different types of MSs can be obtained depending on the synthesis conditions and the surfactant employed [22]. Among them, the Mobile Composition of Matters (MCM) and the Santa Barbara Amorphous (SBA) families are the MSs most widely used [23,24]. Traditionally, these silicas use tetraethyl orthosilicate (TEOS) or sodium silicate (Na_2_SiO_3_) as the silicon source. However, TEOS is an expensive and moisture-sensitive reagent, while Na_2_SiO_3_ is often produced by melting quartz sand and sodium carbonate, which requires a large amount of energy to manufacture, ultimately increasing the cost of the silica. In consequence, to make their synthesis “greener”, Na_2_SiO_3_ can be obtained from RH, which is a natural and cheaper source [19]. Accordingly, different authors have carried out this strategy. For instance, Vanichvattanadecha et al. investigated the effect on surface characteristics and pore morphologies when using RH as the silicon source in the synthesis of MSs [25]. On the other hand, Kamari and Ghorbani used the silica extracted from RH to synthesize a magnetic MCM-41 MS [26]. Likewise, Zhou et al. also used RH to prepare a magnetic MS modified with amino groups, which was successfully applied to the extraction of aflatoxin B1 from aqueous solutions [27].

To date, commercial strong cation exchange (SCX) or mixed-mode (MCX, combination of strong cation exchange and reverse phase interaction) sorbents have shown adequate selectivity for the extraction of alkaloids [28]. Hence, this work proposes the synthesis of an RH-derived SBA-15 MS, which will be functionalized with sulfonic acids (SO₃H) and octadecyl (C18) groups to obtain a MCX-type material. Given the hazards posed by natural plant toxins and the associated regulatory requirements, this study focuses on TAs, PAs, and OAs as priority targets to develop effective control methods in bakery products. For the first time, this material has been applied to the multicomponent solid-phase extraction (SPE) of 29 PAs, TAs, and OAs in gluten-free bread

## 2. Materials and Methods

### 2.1. Chemicals, Solvents and Materials

The solid analytical standards for PAs, except retrorsine, were obtained from Phytolab GmbH & Co. KG (Vestenbergsgreuth, Germany). Atropine, scopolamine hydrobromide, retrorsine, noscapine, and papaverine were purchased from Sigma-Aldrich (St. Louis, MO, USA). The remaining OAs, including morphine, codeine, and thebaine, were obtained from Alcaliber S.A.U. (Madrid, Spain). All alkaloids had a reported purity ≥90%. Stock solutions (at 1000 mg/mL) were prepared in methanol (MeOH). However, stock solutions for europine, europine N-oxide, heliotrine, heliotrine N-oxide, lycopsamine, intermedine, senecionine, seneciphylline, and retrorsine were prepared in a mixture of acetonitrile/dimethyl sulfoxide (ACN/DMSO, 4/1, *v*/*v*). Standard working solutions containing a mixture of all analytes were prepared from the stock solutions at the desired concentrations in MeOH and stored at −20 °C. High-performance liquid chromatography-mass spectrometry (HPLC-MS) grade MeOH and ACN were purchased from Fisher Scientific (Madrid, Spain). HPLC-MS grade formic acid (FA) was obtained from Fluka (Bush, Switzerland), while a 25% ammonia solution (*v*/*v*) was acquired from Merck KGaA (Darmstadt, Germany). DMSO, 37% hydrochloric acid (*v*/*v*), ethanol (EtOH) absolute HPLC grade, 32% ammonia solution (*v*/*v*), and ethyl acetate were acquired from Scharlab (Barcelona, Spain). Isopropanol *(i*-PrOH) was obtained from Merck (Barcelona, Spain), and dichloromethane was purchased from Labkem (Barcelona, Spain). Milli-Q ultrapure water (Billerica, MA, USA) with a resistivity of 18.2 MΩ·cm was used for mobile phase preparation and sample extraction. Samples were filtered using 10 mL syringes and nylon filters (0.45 μm, 25 mm, and 13 mm), also from Scharlab (Barcelona, Spain). SPE comparison was performed using commercial MCX cartridges (Oasis^®^ MCX 6 cc/150 mg/60 μm) from Waters (Milford, MA, USA).

For the synthesis of the sustainable MS sorbent material, RH was acquired from Family Beer (Barcelona, Spain). Pluronic^®^ 123 (P123) (poly (ethylene glycol)-block-poly (propylene glycol)-block-poly (ethylene glycol), EO_20_PO_70_EO_20_), and chloro(dimethyl)octadecylsilane were purchased from Sigma-Aldrich (St. Louis, MO, USA). 30% Hydrogen peroxide (*v*/*v*), sodium hydroxide and synthesis-grade organic solvents (toluene, diethyl ether and ethanol) were obtained from Scharlab (Barcelona, Spain). The 94% (3-mercaptopropyl) triethoxysilane (MPTES) was purchased from Alfa Aesar (Karlsruhe, Germany).

### 2.2. Synthesis and Modification of RH-Derived SBA-15 Silica

#### 2.2.1. Extraction of Silica from RH

Silica was extracted from RH as Na_2_SiO_3_ using an alkaline extraction method based on the procedure described by Liou et al. [29], with slight modifications. First, the RH was thoroughly washed with deionized water and then dried at 100 °C overnight. Subsequently, the dried RH was leached by stirring vigorously at 100 °C for 1 h in 3 M HCl to remove metal impurities. The RH was washed repeatedly with Milli-Q water (Billerica, MA, USA) until the eluent reached a neutral pH, and then it was placed in a muffle furnace and calcined at 600 °C for 6 h. The resulting RH ash silica precursor was stirred in 1.5 M NaOH at 100 °C for 1 h to generate a Na_2_SiO_3_ solution. Finally, this solution was filtered to remove undissolved impurities, and the clear Na_2_SiO_3_ solution (supernatant) was collected and used as a silica precursor for the synthesis of the MS material.

#### 2.2.2. Synthesis and Functionalization of RH-Derived SBA-15 Silica

SBA-15-type MS was synthesized following a conventional hydrothermal method using the Na_2_SiO_3_ obtained from the RH as a source of silicon [29]. Initially, 4.0 g of P123 were mixed with 125 mL of 2.0 M HCl and were continuously stirred at 400 rpm and 35 °C until complete dissolution. Next, 50 mL of 0.3 M Na_2_SiO_3_ were added and stirred at 250 rpm and 35 °C for 20 h. After that, the agitation was stopped, and the temperature was raised to 80 °C for 24 h to carry out the aging process. The resulting solid was washed with Milli-Q water and filtered to remove surfactant residues, then it was dried at 100 °C overnight. Finally, the dry solid was calcined as follows: ramp up for 8.30 h to 500 °C and kept for 12 h at 500 °C. The resultant material, denoted as RH-SBA-15, was functionalized with SO_3_H groups following the procedure described by Gonzalez-Gomez et al. [30]. Next, for bifunctionalisation, 2.5 g of RH-SBA-15-SO_3_H were mixed at 250 rpm with 0.42 g chloro(dimethyl)octadecylsilane in toluene for 24 h at 80 °C. The resulting RH-SBA-15-SO_3_H-C18 was recovered by filtration, washed with different solvents and oven-dried at 70 °C for 24 h.

### 2.3. Characterization of Mesoporous Silica

The material synthesized was characterized by nitrogen adsorption–desorption isotherms, scanning electron microscopy (SEM), transmission electron microscopy (TEM), X-ray diffraction (XRD), elemental analysis (EA), attenuated total reflection Fourier transform infrared spectroscopy (ATR-FTIR) and nuclear magnetic resonance spectroscopy (NMR). Isotherms were measured with a Micromeritics ASAP 2020 analyzer (Micrometrics, Norcross, GA, USA). Before the analysis, 0.2 g of the sample were dried under vacuum and degassed at 90 °C for 10 h in the degassing port of the porosimeter. The Brunauer–Emmett–Teller (BET) method was used to calculate the specific surface area, while the Barrett–Joyner–Halenda (BJH) method was used to obtain the pore size distribution. Surface morphology was examined using a Nova Nano SEM230 (FEG-SEM) (Denton, TX, USA) with an energy-dispersive spectrometry system (EDS). The metallizer used was Leica ACE600 (Wetzlar, Alemania), using a gold target and depositing a layer of less than 5 nm. Before the analysis, the samples were mounted on metal stubs with sticky carbon disks and coated with a 50 nm layer of gold using an SPT-20 sputter coater (300 s, 50 mA). The observations were conducted at an acceleration voltage of 20 kV with magnifications ranging from 70× to 100,000×. A TEM analysis was performed using a JEOL F200 ColdFEG microscope (Akishima, Japan) operating at 200 kV, achieving a resolution of 0.23 nm, with samples mounted on a copper holder. XRD patterns of the material were recorded using a Philips X’Pert MPD/MRD diffractometer (model PW3040/00) operating at 45 kV and 40 mA with Cu Kα radiation (λ = 1.5418 Å). A Thermo Fisher Scientific Flash 2000 analyzer (Waltham, MA, USA) was used to perform an EA of sulfur (% S) and carbon (% C) to estimate the functionalization degree. ATR-FTIR spectra were collected with a PerkinElmer Spectrum Two FT-IR spectrophotometer (Waltham, MA, USA) over the 4000–450 cm^−1^ range to identify primary functional groups before and after functionalization. Additionally, ^13^C cross-polarization magic angle spinning nuclear magnetic resonance (^13^C-CP-MAS-NMR) and ^29^Si pulse decoupling angle magic angle spinning NMR (^29^Si-PDA-MAS-NMR) spectra were recorded on a Bruker Avance III/HD spectrometer at a 400 MHz proton frequency.

### 2.4. Preparation of Gluten-Free Bread Samples

The selection of the gluten-free bread ingredients was based on the possible presence of alkaloids by Commission Regulation (EU) 2023/915 and the European Food Safety Authority (EFSA) [1,2,3,10]. Thus, gluten-free cereals (maize and buckwheat) were selected for the possible presence of TAs, while aromatic herbs and poppy seeds were chosen for the possible occurrence of PAs and OAs, respectively. The gluten-free bread was prepared based on the formulation proposed by Ruiz-Aceituno et al. [31], with slight modifications to adapt the recipe to the objectives of this study, using the following ingredients: vegan and gluten-free maize flour, gluten-free organic buckwheat flour, salt, sugar, gluten-free yeast, 100% natural and gluten-free xanthan gum, gluten-free psyllium husk, extra virgin olive oil (EVOO), aromatic herbs (basil and oregano), and poppy seeds. All ingredients were purchased and stored at room temperature until use. The gluten-free bread preparation carried out is detailed in the Supplementary Material (See Supplementary Information S1).

### 2.5. Extraction by SLE and Purification by SPE with RH-SBA-15-SO_3_H-C18 of Alkaloids from Gluten-Free Bread Samples

PAs, Tas, and OAs were extracted from the prepared gluten-free bread samples by solid–liquid extraction (SLE), following the procedure previously developed by Vera-Baquero et al. [32] with some modifications. A total of 0.5 g of the bread sample was transferred to a 50 mL propylene tube, to which 5 mL of extraction solvent (0.2% FA in water, *v*/*v*) were added. The mixture was subjected to magnetic stirring for 30 min (IKA RCT basic, Staufen, Germany) and centrifuged at 9000 rpm (9146× *g*) for 10 min. The supernatant obtained was recovered and filtered using 0.45 µm nylon filters. Finally, the extract obtained was acidified to pH 1.0 with HCl, and then it was purified and preconcentrated by SPE using RH-SBA-15-SO_3_H-C18 as a sorbent phase.

For SPE, the sorbent material (150 mg of RH-SBA-15-SO_3_H-C18) was packed in polypropylene SPE cartridges sealed at both ends with polyethylene disks. In addition, a nylon filter membrane (0.45 μm) was incorporated at the base of the sorbent bed to prevent its loss during the process. The cartridges were conditioned with 3 mL of Milli-Q water and then equilibrated with 3 mL of acidified water to pH 1.0 with HCl. Subsequently, 5 mL of the sample extract were loaded on the cartridge and vacuum dried for 3 min. Analytes were eluted from the cartridge using 4 mL of 5% ammonia solution in MeOH. The cartridge was vacuum-dried for 3 min. Afterwards, the eluate obtained was evaporated to complete dryness using a double key vacuum line vacuum/nitrogen Schlenk technique at room temperature. The dried eluate was reconstituted in 0.5 mL of mobile phase (0.2% FA in water/0.2% ammonia solution in MeOH, 95/5, *v*/*v*) for its injection into the UHPLC-IT-MS/MS system. This purification procedure allowed a preconcentration factor of 10.

### 2.6. Chromatographic Conditions

Chromatographic separation of the 29 PAs, TAs, and OAs was conducted using a UHPLC system (Dionex UltiMate 3000, Thermo Scientific, Waltham, MA, USA), adapting the conditions optimized in our previous study [32]. A Phenomenex Luna Omega 1.6 μm Polar C18 column (100 × 2.1 mm, Torrance, CA, USA) was used, paired with a SecurityGuard™ protective column containing porous polar C18 sorbent (2 × 2.1 mm, 1.6 μm). Separation was achieved via a gradient elution employing two mobile phases: phase A, consisting of water with 0.2% FA (*v*/*v*), and phase B, MeOH with 0.2% ammonia solution (*v*/*v*). The gradient program was as follows: 0–0.5 min at 5% phase B, 0.5–3 min at 10% phase B, 3–7 min at 25% phase B, 7–9 min at 30% phase B, 9–12 min at 70% phase B, and 12–15 min returning to and holding at 5% phase B, resulting in a total run time of 15 min. The flow rate was set to 0.300 mL/min, with an injection volume of 5 μL. The column temperature was maintained at 30 °C, and the sample tray was cooled to 10 °C.

Detection was performed using tandem mass spectrometry (MS/MS) with an ion trap analyzer (amaZon SL, Bruker, Billerica, MA, USA) operating in positive electrospray ionization (ESI) mode. Multiple reaction monitoring (MRM) mode was used to analyze the target compounds, with instrument settings including a capillary voltage of 4500 V, an end-plate offset of 500 V, nebulizer gas at 20 psi, drying gas at 10 L/min, and a drying temperature of 200 °C. Retention times and mass spectrometry parameters for each analyte are provided in Table S1. Quantification relied on the most abundant product ion from the MS^2^ spectrum of each compound, with additional ions monitored to confirm their identity.

### 2.7. Method Validation

The analytical method developed was validated according to Commission Implementing Regulation (EU) 2023/2783 in terms of method quantification limits (MQLs) and identification (or confirmation), recovery, and precision [11]. Linearity, matrix effects (MEs) and method detection limits (MDLs) were also assessed following the criteria described in SANTE/12682/2019 of EC 401/2006 and in the ICH Q2(R1) guidelines [33,34]. Due to the lack of certified reference materials, validation was carried out using the bread sample prepared. Since this sample contained ingredients that could naturally present the target alkaloids (OAs, TAs, PAs), the validation trials were performed using non-fortified (blank) and fortified samples of this matrix. Fortification of the bread samples was carried out at three concentration levels: low (0.5 μg/kg TAs, 40 μg/kg PAs, and 0.15 mg/kg OAs), medium (5 μg/kg TAs, 400 μg/kg PAs, and 1.5 mg/kg OAs), and high (10 μg/kg TAs, 800 μg/kg PAs, and 3 mg/kg OAs). These levels were selected based on the maximum regulatory limits established for these alkaloids in the food products used as ingredients in the bread sample, as described elsewhere [10]. The corresponding volumes of the standard working solutions prepared in MeOH were added to 0.5 g of bread sample, which were then thoroughly homogenized. A 15 min period was allowed for equilibration at room temperature to completely evaporate the solvent before the SLE procedure. The responses obtained in the fortified samples were corrected with the ones obtained in the non-fortified samples by subtraction for those analytes naturally present in the sample.

Linearity was assessed using matrix-matched calibration curves with six concentration levels for each analyte, and the ME was calculated by comparing the slopes obtained in matrix-matched and solvent-based calibration curves (both expressed in µg/L). Identification was based on method selectivity, using retention times and relative intensities of the precursor and product ions of each analyte obtained on their mass spectra. A minimum of two product ions were required for the confirmation of each analyte. For this purpose, chromatograms and mass spectra obtained from standard solutions, non-fortified samples and fortified samples were compared. Thus, analytes were confirmed in the non-fortified samples when retention times and product ions of the analytes detected in these samples matched those of the standard solutions and the fortified samples.

The method sensitivity was determined in terms of MDL and MQL, representing the lowest analyte concentration detectable and quantifiable, respectively, in the bread sample. These limits were estimated using the slope of the matrix-matched calibration curves and the standard deviation (SD) of the response at the lowest concentration level, as follows: MDL = 3.3 × sd at the lowest concentration level/slope; MQL = 10 × sd at the lowest concentration level/slope. To estimate these values, the SLE procedure and the enrichment factor achieved by SPE (EF 10) were considered. According to Commission Implementing Regulation (EU) 2023/2783, MQLs of these toxins should be ≤2 µg/kg for TAs, ≤10 µg/kg for PAs and ≤500 µg/kg for OAs, based on their regulatory maximum concentration limit set for food products used as ingredients in the bread sample prepared (5 μg/kg, 400 μg/kg and 1.5 mg/kg for TAs, PAs, and OAs, respectively) [11].

Recovery trials were carried out at three concentration levels, indicated above, which were selected based on the regulatory maximum concentration limits established for the toxins in the food products used as ingredients in the bread sample prepared, as described elsewhere [10]. Recovery was calculated using the fortified samples, which were compared with the simulated samples (non-fortified samples subjected to extraction and purification, and fortified afterwards with the analytes at the same concentration before evaporation and HPLC analysis). The results were expressed as the mean recovery of six replicates (*n* = 6). Precision was determined by repeatability (RSDr) and within-laboratory precision (RSDwR) at the same concentration levels as the recovery trials and was expressed as the relative SD percentage (%RSD). RSDr was measured on the same day with 6 sample replicates (*n* = 6), while RSDwR was measured on 3 different days with 3 sample replicates (*n* = 9).

## 3. Results

### 3.1. Characterization of Mesoporous Silicas

**Figure 1:**
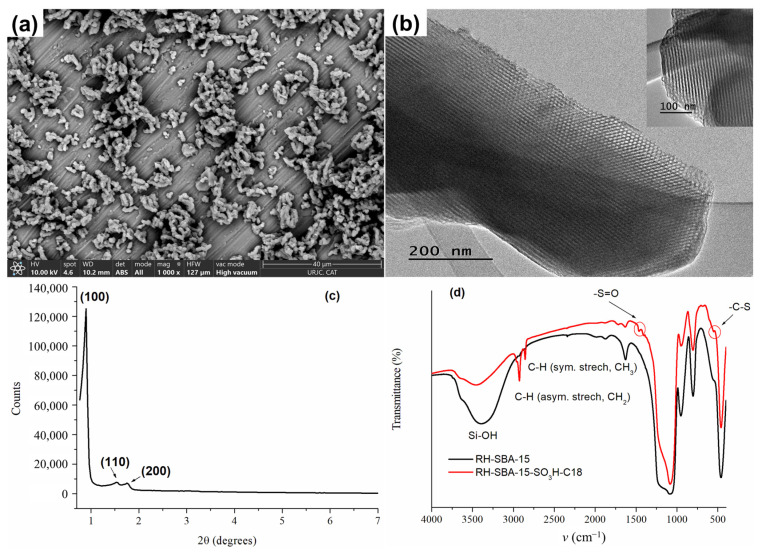
(**a**) SEM; (**b**) TEM images of RH-SBA-15; (**c**) XRD pattern of RH-SBA-15 and (**d**) FT-IR spectra of RH-SBA-15 and RH-SBA-15-SO_3_H-C18.

The morphology and particle size of the bare RH-SBA-15 synthesized were analyzed by SEM and TEM, as illustrated in Figure 1. As shown in Figure 1a, the material appears as particles with a dimension of approximately 1.5 µm on one axis and 800 nm on the other, arranged in agglomerates with a morphology reminiscent of a rope-like structure typical of a conventional SBA-15. TEM images (Figure 1b) show a well-defined and ordered porous structure with a hexagonal honeycomb-like arrangement along the [001] axis and a parallel arrangement of cylindrical pore channels in the [100] direction (Figure 1b inset).

The small-angle XRD data of bare RH-SBA-15 synthesized is displayed in Figure 1c. Three characteristic peaks appeared at the (100), (110), and (200) planes, indicating the formation of a mesoporous structure in the materials. For this material, the XRD pattern displayed a well-resolved pattern at low 2θ values with a very sharp (100) diffraction peak at 0.88° and two well-resolved weak diffraction peaks (110) at 1.52 Å and (200) at 1.76°, characteristic of this type of MS, with a d_100_-spacing value of 100 Å and a unit cell parameter, a_0_, of 116 Å.

**Figure 2:**
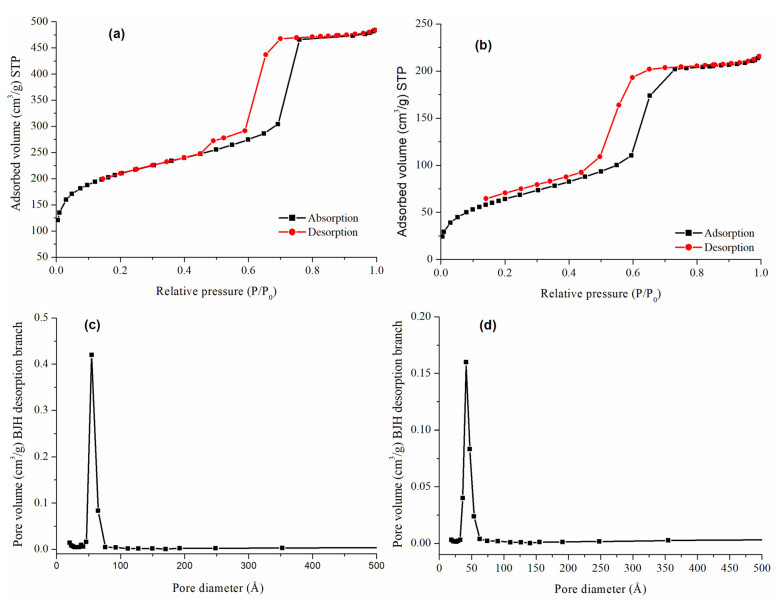
N_2_ adsorption–desorption isotherms of (**a**) RH-SBA-15 and (**b**) RH-SBA-15-SO_3_H-C18. Pore size distribution of (**c**) RH-SBA-15 and (**d**) RH-SBA-15-SO_3_H-C18.

Figure 2 presents the N_2_ adsorption–desorption isotherms for the bare and the functionalized material. Based on the IUPAC classification, type IV isotherms were observed for both materials, with pronounced adsorptions or desorptions at intermediate relative pressures corresponding to the condensation or evaporation of nitrogen in the mesopores. However, the shape of the hysteresis loop was different for each material, probably related to its structure. RH-SBA-15 (Figure 2a) showed mostly an H1-type hysteresis loop with parallel adsorption and desorption branches, characteristic of materials with cylindrical pores of uniform cross-section. Additionally, a transition to an H5-type loop at the end of the curve was observed, indicating the presence of partially blocked pores.

The pore size distribution of the silica was estimated by the BJH method, showing a narrow pore size distribution for RH-SBA-15 (Figure 2c), confirming a uniform mesoporosity of the structure. After bifunctionalization (Figure 2b,d) a decrease in S_BET_ (from 737 to 238 m^2^/g), pore volume (from 0.66 to 0.45 cm^3^/g), and average BJH pore diameter (from 55 to 32 Å) was observed. These changes are attributed to pendant groups on the silica surface, which partially block the adsorption of nitrogen molecules. Moreover, an increase in wall thickness (from 61 to 65 Å) is observed after the functionalization, confirming the presence of ligands within the pores of the material. In addition, the reproducibility of the RH-SBA-15 material was confirmed by obtaining several batches of it, achieving average values of S_BET_: 737 ± 80 m^2^/g, pore volume: 0.7 ± 0.1 cm^3^/g, and pore diameter: 55 ± 1 Å. These values are within the expected range obtained in the conventional synthesis of SBA-15 carried out by our research group over several years (S_BET_: 853 ± 100 m^2^/g; pore volume: 0.9 ± 0.1 cm^3^/g and pore diameter: 53 ± 4 Å).

The successful incorporation of the functional groups (propyl sulfonic and octadecylsilane) into RH-SBA-15 MS was verified using FT-IR, ^29^Si MAS-NMR, and ^13^C MAS-NMR. The FT-IR spectra of the synthesized materials are presented in Figure 1d. The IR patterns of RH-SBA-15 were a typical silica material, showing the Si-O-Si bond at 463 cm^−1^, Si-OH bond at 805 cm^−1^, Si-O bond at 1220 cm^−1^, and -OH bond at 3400 cm^−1^. In addition, the FT-IR spectrum of RH-SBA-15-SO_3_H-C18 shows two peaks at 2929 cm^−1^ (asymmetric stretching, CH_2_) and 2858 cm^−1^ (symmetric stretching, CH_3_) corresponding to the hydrocarbon chains of the functional groups. Furthermore, the band showed at 556 cm^−1^ is related to the C-S stretching mode vibrations, and the band at 1475 cm^−1^ was due to the S=O vibrations. This confirmed the existence of -SO_3_H and -C18 groups in the silica pores.

**Figure 3:**
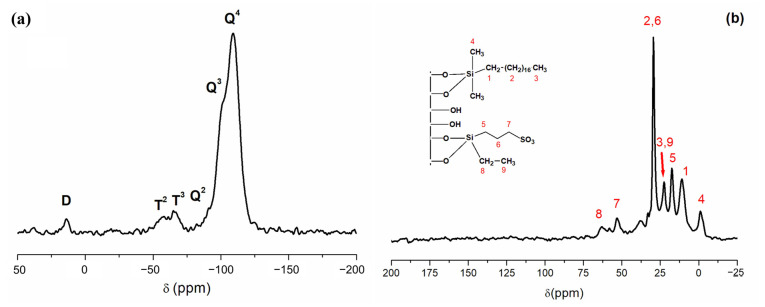
(**a**) ^29^Si PDA-MAS-NMR and (**b**) ^13^C CP-MAS-NMR of the RH-SBA-15-SO_3_H-C18 material.

The ^29^Si-PDA-MAS-NMR spectra in the solid state for RH-SBA-15-SO_3_H-C18 confirmed the formation of the covalent bond between the functional groups and the silanol groups distributed on the RH-SBA-15 surface (Figure 3a). The spectra displayed two primary peaks appearing at −109 and −101 ppm, corresponding to Q^4^ framework silica sites ((SiO)_4_Si) and Q^3^ silanol sites ((SiO)_3_SiOH), respectively. Additionally, a small shoulder appeared at −91 ppm, attributed to the Q^2^ peak ((SiO)_2_Si(OH)_2_). Two other peaks occurred at −64 and −57 ppm, which are assigned to T^3^ ((SiO)_3_Si-R) and T^2^ ((SiO)_2_SiOH–R) sites, respectively. These further confirm the attachment of the organic groups to the silica. Furthermore, the appearance of the signal at 13 ppm, corresponding to D sites associated with the silicon atom in octadecyldimethylsilane, provided additional evidence of successful anchoring of the functional groups to the silica surface.

Significant information regarding the immobilization of the functional groups within the silica surface can also be obtained from the solid state ^13^C-CP-MAS-NMR shown in Figure 3b. The spectra displayed peaks at 29, 22, and 11 ppm, corresponding to the carbon atoms on the C18 group (–(CH_2_)_16_-, –CH_3_, and –CH_2_-, numbered in the figure as (2), (3) and (1), respectively. An additional peak denoted as (4) appeared in the spectrum at −2 ppm due to the carbon atoms on the –CH_3_ of the dimethyloctadecyl group. As can be seen, four signals at 17, 29 and 52 ppm corresponding to the three carbon atoms related to the propyl sulfonic group also appeared, numbered in the figure as (5), (6) and (7). The signal due to methylene (8) of the ethoxy group appeared at 63 ppm, and the signal of the methyl group (9) at 22 ppm. The functional groups attached to the silica (L_0_) were quantified based on the carbon and sulfur content determined through an elemental analysis. The bifunctionalized material (RH-SBA-15-SO_3_H-C18) contains approximately 0.1 mmol/g of C18 ligand and 1.0 mmol/g of SO_3_H groups, estimated from the %C and %S values.

### 3.2. Evaluation of the Adsorption Capacity of RH-SBA-15-SO_3_H-C18 as SPE Sorbent

The selection of the appropriate sorbent in the SPE process is a crucial step for the separation and analysis of chemical compounds, as its efficiency depends on the specific properties of the analytes to be purified. TAs, Pas, and OAs are basic organic molecules that contain functional groups capable of ionizing under acidic conditions, acquiring positive charges. This chemical behavior allows them to be effectively retained by ionic interactions and, in some cases, by hydrophobic interactions. Therefore, commercial MCX sorbents have proven to be efficient for the extraction and purification of these alkaloids [32]. In this work, a RH-derived SBA-15 MS was prepared, taking advantage of its highly ordered structure, high surface area and flexibility to be chemically modified with SO_3_H groups, which allow strong cation-exchange interactions, and C18 chains, to ensure hydrophobic interactions. This resulted in a MCX sorbent that can efficiently retain analytes belonging to all three alkaloid families.

Accordingly, the adsorption capacity of RH-SBA-15-SO_3_H-C18 as an SPE sorbent was evaluated towards the target analytes using standard solutions. For this purpose, an SPE procedure previously described for the extraction of 29 alkaloids from bakery samples using commercial OASIS^®^ MCX cartridges was tested with slight modifications [32]. SPE cartridges were packed with 100 mg of RH-SBA-15-SO_3_H-C18 and were then conditioned and equilibrated with 3 mL of Milli-Q water and 3 mL of 1% HCl in water. Then, 5 mL of a standard solution containing 5 µg/L of TAs, 400 µg/L of PAs, and 1.5 mg/L of OAs in acidified aqueous solution (1% HCl) were loaded on the cartridge. Afterwards, elution was carried out with 3 mL of 5% ammonia solution in MeOH. The results showed that the RH-SBA-15-SO_3_H-C18 sorbent material exhibited a good adsorption capacity towards the analytes, providing recovery values from 70 to 101%, as shown in Figure S1. This high adsorption efficiency can be explained by the structure and functionalization of the material. The high specific surface area of the MS and its uniform pore network maximize the contact between the analytes and the active sites of the sorbent, which contributes to the efficiency of the adsorption process. Likewise, the high degree of functionalization provides multiple electrostatic and hydrophobic interaction sites, thus increasing the retention capacity of the sorbent. The negatively charged SO_3_H provides the sorbent with a SCX capacity. This allows strong electrostatic interactions between the sorbent and the basic analytes presenting protonated amine groups under low pH conditions, which are achieved by acidification with HCl. In addition, the inclusion of C18 groups on the material surface also provides hydrophobic properties to the sorbent, which enhance the retention of non-polar compounds through hydrophobic interactions improving the overall adsorption of the analytes. This combination of ligands makes RH-SBA-15-SO_3_H-C18 a versatile material, capable of interacting through electrostatic and hydrophobic interaction, which are particularly suitable for retaining the different target alkaloids (TAs, PAs and OAs).

Nonetheless, it was observed that the recovery of some analytes remained lower than others (e.g., atropine, codeine, morphine, noscapine, oripavine, and papaverine) (Figure S1). It was assumed that these analytes may require more specific elution conditions or a larger volume of solvent to completely break electrostatic interactions with the SO_3_H groups of the sorbent. Elution with 5% ammonia solution in MeOH raises the basic conditions of the medium, which reduces the protonation of the analytes and allows some of them to return to their neutral form. This change decreases the charges on the analytes, breaking the electrostatic interactions with the SO_3_H groups and allowing their desorption from the sorbent. For these reasons, the effect of the elution volume was investigated by increasing it to 4 mL of 5% ammonia solution in MeOH. It was observed that the recovery values of all the target analytes were above 86% (Figure S1), concluding that a larger solvent volume was necessary to break the electrostatic interactions and release some of the analytes from the sorbent.

### 3.3. Optimization of the Extraction and Purification of Alkaloids from Gluten-Free Bread Samples

Once the adsorption capacity of RH-SBA-15-SO_3_H-C18 MS was successfully confirmed with standards, its application was carried out with the gluten-free bread sample prepared. First, SLE was necessary before the SPE purification. For this purpose, 0.5 g of gluten-free bread, fortified prior to the SLE process with 5 μg/kg of TAs, 400 μg/kg of PAs and 1.5 mg/kg of OAs (medium validation level), were subjected to SLE using 5 mL of extraction solvent. The first solvent tested was the acidified water, since it was the one that had showed the best results in the preliminary adsorption tests carried out with standard solutions, as described in Section 3.2. However, when using this extraction solvent a thick extract (like a paste) was obtained after centrifugation, which impeded the filtration process and caused saturation of the SPE cartridges in the purification process. Therefore, it was necessary to optimize the SLE process by testing different extraction solvents to select the most suitable for the sample matrix.

The SLE is a process influenced by three key mechanisms, (i) the penetration of the solvent chosen into the solid matrix, (ii) the outward diffusivity of the analytes and (iii) the solubility of the analytes in the solvent selected [35]. Among these actions, the adequate selection of the solvent is crucial, since the extraction efficiency depends on the solubility and chemical affinity between the solvent and the analytes, which facilitates their separation and minimizes interferences from other compounds in the matrix. For this reason, the extraction efficiency of the different solvents and solvent mixtures (a mixture of water/MeOH (95:5, *v*/*v*) and EtOH, both acidified with 1% HCl) commonly used for the extraction of these toxins was investigated for the 3 alkaloid families [36,37]. In this instance, unlike with acidified water, clear and easily filterable extracts were obtained. Figure S2 shows the extraction efficiency of the different extraction solvents evaluated, expressed as the recovery (%) of TAs, Pas, and OAs. As can be observed, good recoveries were achieved for the TAs and PAs (73–97%) using acidified water/MeOH (95:5, *v*/*v*), but recoveries for OAs were lower, ranging from 42 to 70% (Figure S2a). On the other hand, good recoveries were also obtained using acidified EtOH as extraction solvent, except in the case of echimidine *N*-oxide, europine, lasiocarpine, senecivernine *N*-oxide, and thebaine, for which recoveries were less than 70% (Figure S2b).

Since good recoveries were not achieved for all the analytes with the solvents tested, the strategy to optimize the SLE procedure was changed bearing in mind the aim of improving the recoveries obtained and the desire to use a solvent as environmentally friendly as possible. Consequently, the influence of the acid additive used in the acidified extraction process was investigated. The type of acid used can significantly influence the extraction efficiency and selectivity of the analytes. For example, strong acids such as HCl can improve the extraction by favoring the ionization of the analytes, which facilitates their solubility in aqueous solutions. However, this can also result in thicker and more viscous extracts, complicating filtration. On the other hand, milder acids, such as FA, represent a less aggressive alternative, as they tend to reduce the viscosity of the extract, making easier its subsequent filtration and purification. Thus, different concentrations of FA (0.2% and 1%) in the water/MeOH mixture (95:5, *v*/*v*) were investigated. As shown in Figure S3a, good recoveries were achieved in all cases, with recoveries from 72 to 100% using water acidified with 0.2% FA and between 72 and 97% for water acidified with 1% FA. Therefore, replacing HCl with FA improved the extraction efficiency. Likewise, no big differences were observed between the two concentrations of FA tested, so a concentration of 0.2% FA was chosen to save reagent costs. Moreover, to make the SLE process more environmentally friendly, the MeOH was removed from the extraction solvent, so 0.2% FA in water was tested as the extraction solvent. The results obtained are presented in Figure S3b, showing good recoveries (73–100%) for all analytes, so this last solvent was chosen for the SLE procedure.

**Figure 4:**
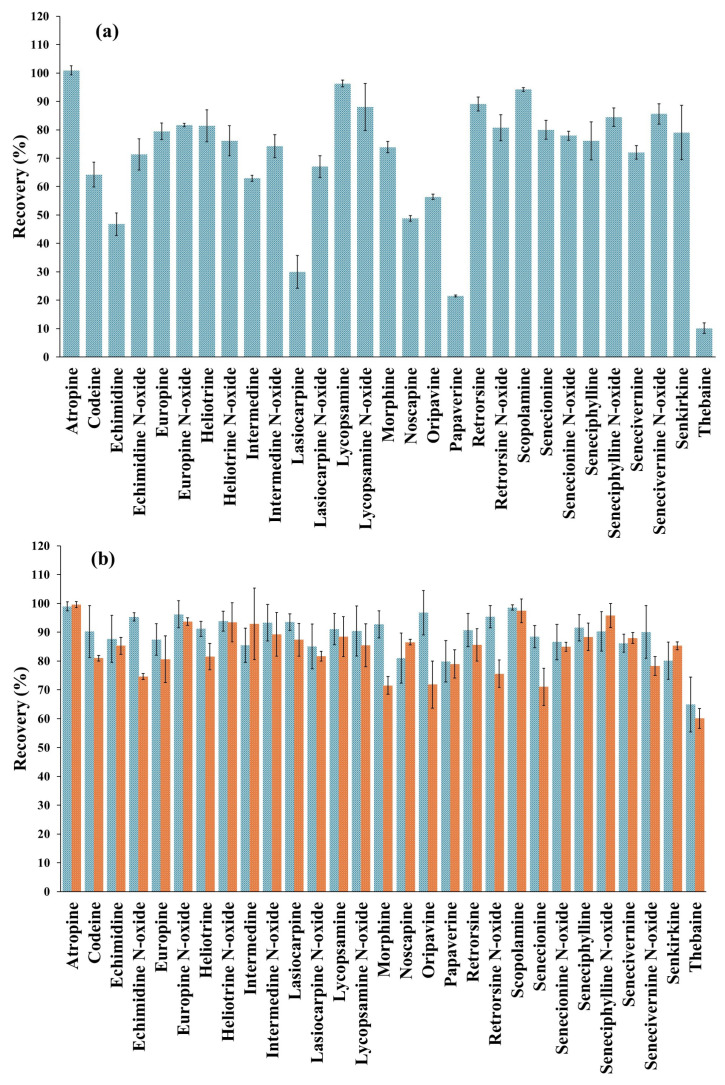
(**a**) Recovery values (% ± SD) obtained from the solid-phase extraction (SPE) analysis of a gluten-free bread sample, fortified with the target analytes (5 μg/kg tropane alkaloids, 400 μg/kg pyrrolizidine alkaloids and 1.5 mg/kg opium alkaloids) using 100 mg of RH-SBA-15-SO_3_H-C18 sorbent, using the following SPE conditions: conditioning with 3 mL of Milli-Q water, equilibration with 3 mL of 1% HCl in water, loading 5 mL of acidified sample extract, vacuum drying for 3 min, elution with 4 mL of 5% ammonia solution in MeOH and vacuum drying for 3 min. (**b**) Recovery values (% ± SD) obtained from the SPE analysis of gluten-free bread sample, fortified with the target analytes (5 μg/kg tropane alkaloids, 400 μg/kg pyrrolizidine alkaloids and 1.5 mg/kg opium alkaloids) using 150 mg of RH-SBA-15-SO_3_H-C18 (blue) and OASIS^®^ MCX (orange) cartridges, under the SPE conditions described above. Error bars represent the standard deviation of sample replicates (*n* = 3).

Once the extraction solvent was selected for the SLE, the sample extract obtained was acidified to pH 1.0 with HCl and purified by SPE using 100 mg of RH-SBA-15-SO_3_H-C18 MS. However, the recovery values obtained with the sample extract were significantly lower than those obtained with the standard solutions, ranging from 10 to 101% (Figure 4a). To improve these results, a second elution step in the SPE was tested by using an additional 2 mL of different solvents, including MeOH, MeOH/water (50:50, *v*/*v*), ACN, *i-*PrOH, ethyl acetate and dichloromethane. This approach aimed to evaluate whether the low recoveries observed were due to insufficient elution volume or whether alternative solvents with different polarities could disrupt the interactions between the analytes and the C18 groups of the sorbent. Nonetheless, with all these solvents, the average recovery obtained in the second elution cycle ranged from 0 to 2% for all analytes, suggesting that the problem was not a lack of elution volume or of solvent polarity. Therefore, as an alternative measure, the sorbent amount packed into the cartridge was increased to 150 mg, and the SPE procedure was carried out under the same conditions as described above. As shown in Figure 4b, the recovery values significantly improved by increasing the amount of sorbent, ranging from 65 to 99%. Only thebaine showed a recovery value below the validation range (70–120%) [33,34]. Nevertheless, this amount of sorbent was selected for the proposed method. To avoid the use of larger amounts of material, which could compromise the sustainability of the method, it was taken into account that, in the case of thebaine, the determined concentration must be corrected using the obtained recovery factor.

Finally, the efficiency of the RH-SBA-15-SO_3_H-C18 sorbent was compared under the same experimental conditions with the widely used commercial sorbent OASIS^®^ MCX (150 mg), which, as explained above, has equivalent functionalities. The results obtained with the commercial OASIS^®^ MCX cartridge showed recovery values between 60% and 100%, which are very similar to those obtained with the RH-SBA-15-SO_3_H-C18 sorbent (Figure 4b). Therefore, SBA-15-RH-SO_3_H-C18 MS can be used as a renewable and viable alternative to commercial sorbents due to its versatility and customized approach for the extraction of the target alkaloids. However, while these recovery rates highlight its analytical potential, further studies are needed to investigate the long-term stability, cost-effectiveness, and scalability of RH-SBA-15-SO_3_H-C18 to fully assess its suitability for industrial applications.

In conclusion, the methodological approach developed in this study offers an innovative alternative to other extraction procedures such as SLE or SLE-SPE (Table 1), being remarkable for using a notably smaller amount of bread sample, only 0.5 g, in contrast to previous research using up to 15 g [38,39,40,41,42]. Another advantage is the use of water as an extraction solvent, a more sustainable, less toxic, and safer choice compared to solvents like MeOH, ACN, or chloroform commonly used in similar studies (Table 1), while also achieving a substantial reduction in the required solvent volume compared to the up to 45 mL reported in other methods [38,39,40,41,42,43]. Lastly, a notable benefit is the use of an adsorbent whose silicon source is derived from RH, an agro-industrial waste, positioning it as a practical and effective alternative to commercial adsorbents for detecting natural toxins in food [32,43].

### 3.4. Method Validation

The results obtained from the validation of the methodology proposed for the determination of 29 alkaloids in gluten-free bread samples are presented in Table 2. The calibration curves achieved coefficients of determination (R^2^) higher than 0.999 within the linear concentration ranges evaluated, demonstrating good linearity of the method according to the reference validation guidelines documents [33,34]. In addition, the deviation of the slopes of the calibration curves was calculated for different days (*n* = 3) to ensure reproducibility, obtaining RSD values between 1 and 18%. In terms of sensitivity, the method LODs and LOQs for the target alkaloids ranged between 0.12 and 1.56 μg/kg and 0.38–5.43 μg/kg, respectively (Table 2). These values demonstrated the capability of the method to detect and quantify the target analytes at concentration levels well below the regulatory limits set for these alkaloids in foodstuffs [10,11]. On the other hand, the proposed method did not show ME, as all values ranged between −13% and 11%, not exceeding in any case ±20% [33]. Therefore, the purification procedure with RH-SBA-15-SO_3_H-C18 MS effectively mitigated matrix interferences, ensuring suitable clean-up that helped to achieve an accurate detection and identification of the target analytes in the gluten-free bread samples. Therefore, solvent-based calibration curves (Table S2) could be used for accurate quantification, which simplifies the procedure. The precision and accuracy of the method were assessed at three different concentration levels. The accuracy in terms of recovery showed suitable average values ranging from 70 to 97% (Table 2), which are within the 70–120% range established by the validation guidelines [33,34]. Likewise, satisfactory results were obtained for RSDr and RSDwR precision at the 3 concentration levels, as the RSD values in both cases were lower than 20% (Table 2), as established in the validation criteria [33,34]. Therefore, the proposed method showed to meet all the established validation criteria, demonstrating its reliable analytical performance and reinforcing its feasibility for routine analytical use. Thus, it can be effectively employed to accurately detect and quantify TAs, Pas, and OAs in gluten-free bread samples.

To the best of our knowledge, there is limited literature on the development of analytical methods designed to simultaneously analyze TAs, PAs, and OAs in gluten-free bread products (Table 1). Similarly, few validated methods exist specifically for detecting these compounds in bread. For instance, Marín-Sáez et al. developed a method based on HPLC-QqQ-MS/MS to analyze only TAs, using SLE on a homemade bread sample. This method achieved recoveries ranging from 75% to 101%, with repeatability, assessed as intra-day precision through RSD, ranging between 1% and 13% across all cases [39].

Compared to these earlier studies (Table 1), our proposed methodology stands out by delivering superior validation parameters, particularly very low LOQs, a near absence of ME, and good recoveries (ranging from 63% to 100% at the medium validation level). This demonstrates that our approach is an effective and reliable tool for the simultaneous monitoring of these three alkaloid families.

### 3.5. Application of the Validated Method to the Analysis of Gluten-Free Bread

To confirm the applicability of the method, the proposed methodology was applied to the analysis of the non-fortified gluten-free bread sample prepared in the laboratory, which included ingredients likely to be contaminated with the target alkaloids. Three replicates of this sample were analyzed in triplicate. Matrix-matched calibration curves were performed for each analyte for their quantification in the gluten-free bread samples, although no ME were detected during method validation, as previously explained. Figure S4 shows the average content of the alkaloids naturally found in the gluten-free bread sample analyzed. No contamination with PAs was found in the sample, despite the fact that it contained some ingredients susceptible to being contaminated with these alkaloids, such as oregano or maize [45,46,47,48]. Nonetheless, the proportion of oregano added during bread preparation was very small. In addition, levels of PAs found in cereal flours are usually not very high [45,46,47,48].

**Figure 5:**
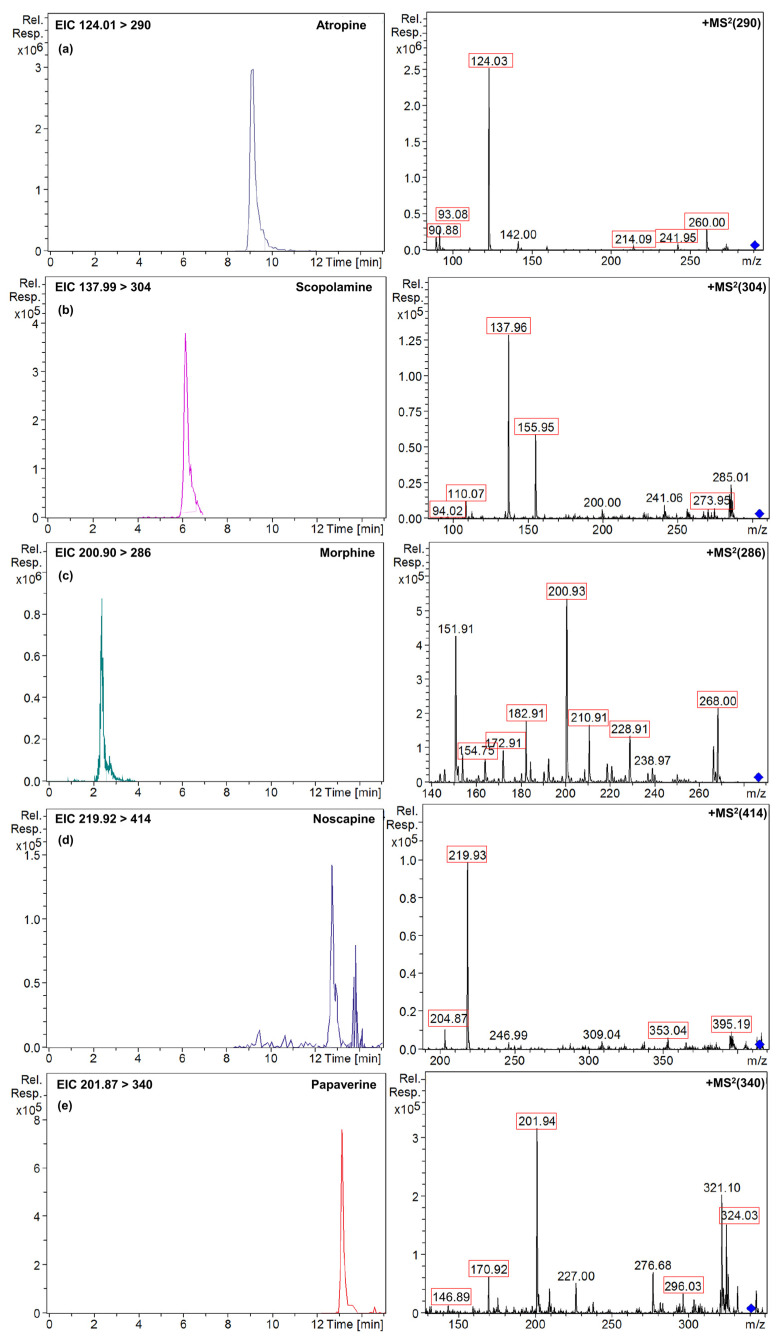
Extracted ion chromatograms and mass spectra (MS^2^) of (**a**) atropine; (**b**) scopolamine; (**c**) morphine; (**d**) noscapine; and (**e**) papaverine naturally found in gluten-free bread sample. Red squares indicate the product ions se for quantification and confirmation of the target analytes (Table S1). The blue symbol corresponds to the precursor ion of each analyte.

Figure 5 presents the extracted ion chromatograms (EICs) and mass spectra (MS^2^) of the analytes detected in the naturally contaminated gluten-free bread sample, confirming the presence of TAs and OAs through their MS^2^ spectra. Additionally, the MS^2^ spectra of the peaks detected in the sample closely match those shown in Figure S5, which displays the MS^2^ spectra of the standard solutions, exhibiting differences in ion ratios below 30% and a variation time of ±0.1 min, further supporting the reliable identification of these alkaloids [33,34]. Complementary to this, Figure S4 provides the total content (μg/kg) of TAs and OAs quantified in the gluten-free bread sample, offering a clear overview of the contamination levels detected.

Regarding the TAs, the sample contained atropine and scopolamine at concentrations of 272 ± 6 and 37.7 ± 0.2 µg/kg, respectively (Figure S4). The gluten-free bread sample was made from maize flour and buckwheat flour, which are products highly susceptible to containing TAs [5]. It is worth noting that the concentrations found for these toxins are very high, as they exceed the maximum concentration limits legislated for milling products of maize and buckwheat, which are 5 and 10 µg/kg, respectively, as the sum of atropine and scopolamine [10]. Nevertheless, according to Commission Regulation (EU) 2021/1408 of 27 August 2021, amending Regulation (EC) No 1881/2006 regarding the maximum levels of TAs in certain foodstuffs [8], processed cereal-based foods containing maize or its derived products, lawfully placed on the market before 1 September 2022, may remain on the market until their date of minimum durability or expiration date, which is the case for the flours used in this work. These high concentrations of TAs pose potential health risks, as TAs are known to cause acute toxicity, including anticholinergic effects such as blurred vision, dry mouth, and, in severe cases, hallucinations or respiratory distress, particularly in vulnerable populations [3]. The primary contamination pathway for TAs in food is linked to the co-harvesting of crops with toxic plants, such as Datura species, which share similar maturation cycles with certain food crops like cereals and pseudocereals. The seeds of these toxic plants, often similar in shape and size to those of food crops, are the main source of contamination, and their accidental inclusion is facilitated by mechanized harvesting practices that rapidly collect both crops and weeds. Moreover, the reluctance to use phytosanitary products and the growing trend toward organic products increase the likelihood of natural toxin contamination. To mitigate these risks, food safety stakeholders could adopt good agricultural practices, such as implementing stricter field monitoring to prevent co-harvesting with toxic plants and improving seed sorting techniques during the harvesting and storage. Additionally, various pre-treatment and food processing techniques, such as washing, sorting, or dehulling, could help reduce the content of these toxins [1,2,3,4].

On the other hand, the gluten-free bread sample contained 5% poppy seeds among its ingredients, which may be a contamination source for OAs. However, only three OAs were detected and quantified in the sample (Figure 5 and Figures S4 and S5), including morphine at 0.062 ± 0.001 mg/kg, noscapine at 0.0025 ± 0.0001 mg/kg, and papaverine at 0.010 ± 0.001 mg/kg, at levels that were below the maximum concentration limit legislated for OAs in bakery products containing poppy seeds or derivatives, which is 1.5 mg/kg morphine equivalents [10]. The baking process may also influence alkaloid levels, as thermal degradation could potentially lower their concentrations; however, in the analyzed bread samples, poppy seeds were distributed both on the surface and within the dough. Studies suggest that thermal degradation might be more significant for seeds on the surface due to higher exposure to heat, but the dough’s protective effect may have limited this degradation, allowing TAs and OAs to remain present [43]. This aspect warrants further investigation to determine how the temperature or fermentation processes in bakery products influence the degradation of these compounds and whether such processes can ensure consumer safety. Future studies should focus on a detailed risk assessment to better understand the health implications of these concentrations and to develop comprehensive mitigation strategies.

## 4. Conclusions

RH is an agri-food waste that can be considered a promising natural resource for the preparation of sustainable MS materials, standing out as an interesting approach for the circular economy. RH was successfully used as a renewable and alternative silica source to Na_2_SiO_3_ to obtain an SBA-15 type MS, which was then bifunctionalized with SO_3_H and C18 groups. Accordingly, RH-SBA-15-SO_3_H-C18 MS was effectively synthesized and characterized for the first time showing interesting properties for its use as MCX sorbent material in sample preparation. The analyses confirmed the ordered mesoporous structure, high specific surface area, uniform pore distribution and other outstanding properties of the material. Likewise, it was confirmed that the functional groups were correctly attached to the silica surface, providing the material with specific mixed-mode chemical properties, such as simultaneous SCX and hydrophobic interactions provided by the SO_3_H and C18 groups, respectively. Thus, the material was suitable for its use as an SPE sorbent for the extraction and purification of TAs, Pas, and OAs. Its effectiveness as an SPE sorbent was tested for the extraction and purification of these natural toxins from gluten-free bread samples showing good analytical performance, highlighting good recovery values and the absence of matrix interferences. This demonstrates the purification and cleaning capacity of the sorbent, which are two crucial aspects in sample preparation. Moreover, the efficiency of RH-SBA-15-SO_3_H-C18 MS was compared with a MCX commercial sorbent with equivalent functionalities showing very similar results, which confirmed that this material could be used as a renewable and viable alternative for the extraction of the target alkaloids in food samples. The feasibility of the RH-SBA-15-SO_3_H-C18 sorbent was also confirmed through its application to the analysis of the gluten-free bread sample, which revealed the natural contamination of this matrix with TAs and OAs, in some cases exceeding the maximum concentration limits established in legislation, which may pose a potential risk to public health. These results, therefore, underline the need to simultaneously monitor the occurrence of these toxins in food, thus contributing to ensuring food safety and consumer protection.

## Figures and Tables

**Table 1:** Evaluation of the proposed approach against the existing techniques used in bakery products.

Bakery Products	Analytes	Sample Preparation	Analytical Technique	LOQ *	Recovery (%)	RSD * (%)	ME * (%)	Reference
Baking mixes	Morphine, codeine, papaverine and noscapine	SLE Sample: 10 g Extraction solvent: 30 mL methanol with 0.1% acetic acid	LC/MS/MS	0.07–0.3 ^a^ mg/kg	N.S	7.4–9.0	N.S	[38]
Bread	Atropine, scopolamine and 15 other tropane alkaloids	SLE Sample: 3 g Extraction solvent: 30 mL methanol:water (2:1, *v*/*v*) with 0.5% acetic acid	LC-Orbitrap-MS	N.S	75–101%	1–13%	N.S	[39]
Muffin	Morphine, codeine and thebaine	SLE Sample: 15 g Extraction solvent: 45 mL acetonitrile/water (80/20; *v*/*v*) with 0.1% formic acid	UPLC−MS/MS	0.6–2.3 pg	N.S	N.S	N.S	[40]
Muffins and poppy rolls (baking topping)	Morphine, codeine, thebaine, noscapine and papaverine	SLE Sample: 400 mg Extraction solvent: 1 mL chloroform: isopropanol (90:10, *v*/*v*) at pH 3.5	HPLC−IT−MS	10 ng/mL	N.S	1.1–3.6	N.S	[41]
Muffin batter	Noscapine	SLE Sample: 15 g Extraction solvent: 45 mL acetonitrile/water (80/20; *v*/*v*) with 0.1% formic acid	UPLC−MS/MS	3.6 mg/kg	N.S	N.S	N.S	[42]
Gluten-Free Corn Breadsticks	Morphine, codeine, thebaine, papaverine, noscapine, atropine, scopolamine and anisodamine	SLE Sample: 2.5 g Extraction solvent: 20 mL of methanol with 0.1% acetic acid	HPLC-DAD	1.01–8.16 mg/kg	96–110	1–18	80–109	[43]
Bakery products (bread, slice bread, bread toasts, breadsticks, crackers and biscuits)	21 pyrrolizidine, 2 tropane and 6 opium alkaloids	SLE/SPE Sample: 0.5 g Extraction solvent: 5mL water with 1% HCl Purification: 150 mg Oasis MCX	UHPLC-IT-MS/MS	0.38–5.43 µg/kg	77–98	1–10	−19–0	[32]
Homemade gluten-free ground poppy seed crackers	Morphine, codeine, thebaine, papaverine, noscapine and oripavine	SLE/SPE Sample: 0.5 g Extraction solvent: 8 mL acidified water (pH 1.0 HCl) Purification: 50 mg SBA-15- SO_3_H-CN	UHPLC-TQ-MS/MS	0.6–1.1 μg/kg for TAs 0.06–0.46 mg/kg for OAs	79–107	2–17	−80–15	[44]
Bread	21 pyrrolizidine, 2 tropane and 6 opium alkaloids	SLE/SPE Sample: 0.5 g Extraction solvent: 5 mL water with 0.2% formic acid Purification: 150 mg RH-SBA-15-SO_3_H-C18	HPLC-IT-MS/MS	0.38–5.43 µg/kg	63–100	1–11	−11–11	This work

* LOQ: limit of quantification of the method; RSD: relative standard deviation; ME: matrix effects; SLE: solid–liquid extraction; SPE: solid-phase extraction; LC: liquid chromatography; HPLC: High-Performance Liquid Chromatography; UPLC: ultraperformance liquid chromatography; UHPLC: Ultra-High-Performance Liquid Chromatography; IT: Ion Trap; MS: mass spectrometry; TQ: triple quadrupole; N.S: not shown. ^a^ The value showed is the LOD.

**Table 2:** Analytical parameters of the method proposed for the analysis of pyrrolizidine, tropane, and opium alkaloids in gluten-free bread samples.

Analytes	Linear Range (µg/kg)	Matrix-Matched Calibration (R^2^)	Recovery (% ± SD)			Mean Recovery (% ± SD)	Repeatability Precision (RSD%)			Within-Laboratory Precision (RSD%)			MDL * (µg/kg)	MQL * (µg/kg)	ME (%)
			Low ^a^	Medium ^b^	High ^c^		Low ^a^	Medium ^b^	High ^c^	Low ^a^	Medium ^b^	High ^c^			
Atropine	1.00–300	y = 296,785x + 3106 (0.999)	97 ± 1	100 ± 1	94 ± 2	97 ± 3	1	1	2	4	3	3	0.12	0.38	4
Codeine	2.00–3000	y = 99,111x + 2106 (0.999)	82 ± 8	85 ± 2	88 ± 7	85 ± 3	10	3	8	9	8	7	0.42	1.38	−12
Echimidine	1.00–300	y = 285,245x − 131,526 (0.999)	94 ±2	83 ± 5	94 ± 4	90 ± 6	2	6	4	4	7	7	0.28	0.92	4
Echimidine *N*-oxide	2.00–300	y = 454,369x − 17,066 (0.999)	90 ± 4	91 ± 6	87 ± 6	90 ± 2	5	6	7	5	7	7	0.47	1.56	−10
Europine	6.00–300	y = 212,879x + 571,075 (0.999)	86 ± 6	78 ± 4	82 ± 8	82 ± 4	7	6	9	9	5	10	1.63	5.43	−12
Europine *N*-oxide	6.00–300	y = 362,953x + 400,796 (0.999)	98 ± 2	83 ± 2	82 ± 8	87 ± 9	2	2	10	5	8	8	1.56	5.20	−3
Heliotrine	1.00–300	y = 226,107x + 44,518 (0.999)	87 ± 7	79 ± 7	98 ± 4	88 ± 9	8	9	4	8	6	5	0.26	0.87	−2
Heliotrine *N*-oxide	1.00–300	y = 280,627x + 33,815 (0.999)	93 ± 2	88 ± 3	93 ± 7	91 ± 3	3	4	7	4	4	7	0.50	1.67	1
Intermedine	2.00–300	y = 151,285x + 64,688 (0.999)	87 ± 3	79 ± 6	89 ± 7	85 ± 5	3	8	8	2	10	7	0.52	1.72	11
Intermedine *N*-oxide	2.00–300	y = 168,591x + 256,072 (0.999)	93 ± 4	90 ± 8	88 ± 4	90 ± 3	5	9	5	5	8	6	0.52	1.73	−5
Lasiocarpine	2.00–300	y = 587,404x − 620,362 (0.999)	94 ± 9	85 ± 7	95 ± 9	92 ± 6	10	8	9	4	4	8	0.37	1.22	4
Lasiocarpine *N*-oxide	2.00–300	y = 586,066x − 123,946 (0.999)	92 ± 3	93 ± 3	89 ± 5	91 ± 2	3	3	6	3	9	5	0.37	1.24	−6
Lycopsamine	2.00–300	y = 136,734x + 61,960 (0.999)	96 ± 2	90 ± 5	89 ± 5	92 ± 4	2	6	6	5	9	6	0.43	1.44	−7
Lycopsamine *N*-oxide	2.00–300	y = 169,105x + 260,741 (0.999)	93 ± 2	93 ± 4	90 ± 5	92 ± 2	2	5	6	3	10	5	0.39	1.30	−9
Morphine	1.00–3000	y = 79,799x + 459,065 (0.999)	100 ± 4	73 ± 5	79 ± 3	84 ± 14	4	7	4	10	10	10	0.25	0.83	−13
Noscapine	1.00–3000	y = 5,666,667x – 5,668,012 (0.999)	74 ± 4	74 ± 4	88 ± 9	79 ± 8	5	6	10	3	5	7	0.13	0.43	−6
Oripavine	2.00–3000	y = 25,775x + 33,223 (0.999)	88 ± 7	85 ± 3	88 ± 3	87 ± 2	8	3	4	5	10	8	0.42	1.38	0
Papaverine	1.00–3000	y = 894,000x + 609,973 (0.999)	81 ± 4	77 ± 3	77 ± 4	78 ± 3	5	4	5	3	8	5	0.23	0.78	0
Retrorsine	1.00–300	y = 145,873x + 54,022 (0.999)	95 ± 5	77 ± 3	92 ± 3	88 ± 9	6	3	3	6	6	2	0.25	0.84	−10
Retrorsine *N*-oxide	2.00–300	y = 35,152x + 85,205 (0.999)	92 ± 3	80 ± 4	91 ± 5	88 ± 7	4	6	5	4	9	6	0.52	1.73	−4
Scopolamine	1.00–300	y = 116,194x + 497,310 (0.999)	99 ± 2	98 ± 3	90 ± 3	96 ± 5	2	3	3	2	1	4	0.18	0.59	−9
Senecionine	2.00–300	y = 349,644x − 400,100 (0.999)	93 ± 1	89 ± 6	99 ± 2	94 ± 5	1	7	2	2	5	8	0.44	1.47	−7
Senecionine *N*-oxide	2.00–300	y = 51,515x + 39,822 (0.999)	92 ± 6	76 ± 4	91 ± 4	87 ± 9	6	6	5	6	5	5	0.38	1.27	−12
Seneciphylline	2.00–300	y = 122,051x + 343,330 (0.999)	82 ± 6	84 ± 7	93 ± 4	86 ± 6	8	9	4	7	5	3	0.37	1.24	−2
Seneciphylline *N*-oxide	4.00–300	y = 50,238x + 17,004 (0.999)	85 ± 3	79 ± 6	93 ± 5	86 ± 7	4	7	6	6	7	7	1.04	3.46	−9
Senecivernine	2.00–300	y = 314,944x + 81,413 (0.999)	94 ± 4	84 ± 6	90 ± 5	89 ± 5	4	7	5	6	5	5	0.37	1.23	−3
Senecivernine *N*-oxide	2.00–300	y = 64,911x − 7928 (0.999)	84 ± 6	79 ± 6	89 ± 5	84 ± 5	7	8	6	7	8	6	0.43	1.44	−5
Senkirkine	2.00–300	y = 139,817x − 89,539 (1)	91 ± 5	93 ± 7	92 ± 7	92 ± 1	5	7	7	10	8	8	0.39	1.31	−6
Thebaine	2.00–3000	y = 49,133x + 30,837 (0.999)	81 ± 3	63 ± 7	67 ± 1	70 ± 10	3	11	2	5	9	5	0.52	1.74	0

* MDL: method detection limits and MQL: method quantification limits estimated as 3.3× standard deviation at the lowest calibration level/slope and 10× standard deviation at the lowest calibration level/slope, respectively (concentration expressed in the sample before extraction). Matrix effect (ME) = [(slope matrix-matched calibration/slope solvent-based calibration) − 1] × 100. Accuracy (mean recovery obtained from six sample replicates, *n* = 6) and precision were obtained by spiking samples at three known concentration levels. Repeatability: six sample replicates injected in triplicate on the same day (*n* = 6); within-laboratory precision: three sample replicates injected in triplicate throughout three different days (*n* = 9). ^a^ Low (0.5 μg/kg of tropane alkaloids, 40 μg/kg of pyrrolizidine alkaloids and 0.15 mg/kg of opium alkaloids), ^b^ medium (5 μg/kg of tropane alkaloids, 400 μg/kg of pyrrolizidine alkaloids and 1.5 mg/kg of opium alkaloids) and ^c^ high (10 μg/kg of tropane alkaloids, 800 μg/kg of pyrrolizidine alkaloids and 3 mg/kg of opium alkaloids).

## Data Availability

The original contributions presented in this study are included in the article/Supplementary Material. Further inquiries can be directed to the corresponding authors.

## Supplementary Materials

The following supporting information can be downloaded at: https://www.mdpi.com/article/10.3390/foods14071156/s1, Supporting Information S1; Table S1: retention time and mass spectrum parameters for the analysis of 29 alkaloids using the UHPLC-IT-MS/MS method described in this work; Table S2: instrumental validation parameters for UHPLC-IT-MS/MS analysis of tropane, pyrrolizidine and opium alkaloids; Figure S1: recovery values (% ± SD) obtained from the solid-phase extraction (SPE) analysis of standard solutions (5 μg/kg tropane alkaloids, 400 μg/kg pyrrolizidine alkaloids and 1.5 mg/kg opium alkaloids) in acidified water (1% HCl) using 100 mg of RH-SBA-15-SO3H-C18 as sorbent and different elution volumes (3 and 4 mL). SPE conditions: conditioning with 3 mL Milli-Q water, equilibration with 3 mL of 1% HCl in water, loading 5 mL of standard solution, vacuum drying for 3 min, elution with 3 or 4 mL of 5% ammonia solution in methanol, and vacuum drying for 3 min. Error bars represent the standard deviation of sample replicates (*n* = 3); Figure S2: recovery values (% ± SD) obtained for solid–liquid extraction (SLE) of tropane, pyrrolizidine and opium alkaloids from the gluten-free bread sample fortified with the target analytes at 5 μg/kg, 400 μg/kg s and 1.5 mg/kg, respectively, using (a) water/methanol (95:5 *v*/*v*) with 1% HCl and (b) in ethanol with 1% HCl as extraction solvents. SLE conditions: 0.5 g sample, 5 mL extraction solvent, 30 min magnetic stirring, 10 min centrifugation at 9000 rpm, filtration and injection into the UHPLC-IT-MS/MS system. Error bars represent the standard deviation of sample replicates (*n* = 3); Figure S3: recovery values (% ± SD) obtained for solid–liquid extraction (SLE) of tropane, pyrrolizidine and opium alkaloids from the gluten-free bread sample fortified with the target analytes at 5 μg/kg, 400 μg/kg and 1.5 mg/kg, respectively, using (a) water/methanol (95:5 *v*/*v*) with 0.2 and 1% formic acid and (b) water with 0.2% formic acid as extraction solvents. SLE conditions: 0.5 g sample, 5 mL extraction solvent, 30 min magnetic stirring, 10 min centrifugation at 9000 rpm, filtration and injection into the UHPLC-IT-MS/MS system. Error bars represent the standard deviation of sample replicates (*n* = 3); Figure S4: total content (μg/kg) of tropane and opium alkaloids quantified in the gluten-free bread sample. Figure S5: Extracted Ion Chromatograms (EICs) and mass spectra (MS^2^) of atropine, scopolamine, morphine, noscapine, and papaverine in a standard solution. At the top of each EIC, the isolated precursor ion (*m*/*z*) is indicated on the left along with its extracted product ion to obtain the MS^2^, while the name of the corresponding analyte is indicated on the right. At the top of each mass spectrum, the isolated precursor ion (*m*/*z*) is indicated on the right along with the ionization mode.
