# Supplementary material for: Application of Rice Husk-Derived SBA-15 Bifunctionalized with C18 and Sulfonic Groups for Solid-Phase Extraction of Tropane, Pyrrolizidine, and Opium Alkaloids in Gluten-Free Bread
Source: Foods. 2025 Mar 26;14(7):1156. doi: 10.3390/foods14071156 (PMC11988533; doi:10.3390/foods14071156)
Supplement: Supplementary file 1 [file foods-14-01156-s001.zip › foods-3528571-supplementary.pdf]

Supporting Information S1

The gluten-free bread was prepared according to the following procedure: the dry ingredients (205.4 g maize and 99.8 g buckwheat flours, 18.0 g sugar, aromatic herbs (1.3 g oregano and 1.3 g basil) and 16.1 g poppy seeds) were weighed and placed in a bowl. Poppy seeds were first pre-crushed using an electric grinder at a speed of 28,000 rpm (52,685 x g, relative centrifugal force, analytical mill A11 Basic, IKA, Staufen, Germany) to ensure an even distribution of poppy seeds in the bread dough. All these ingredients were mixed using a kneader-mixer (Alphamix 2 Matfer Bourgeat mixer, Longny-au-Perche, France) operating at speed 1 for 1 min. Subsequently, liquid ingredients (377.9 g water and 15.9 g EVOO) were weighed and incorporated into the dry ingredients. Hydrocolloids, which included xanthan gum (5.5 g) and psyllium husk (1.0 g), were then added, followed by the yeast (3.2 g) and the salt (4.8 g), in that order. For the combination of all the ingredients, the kneader-mixer was operated at speed 2 for 6 min to integrate all components and obtain a homogeneous dough. Once the dough was obtained, it was allowed to rest for 15 min before being transferred to the baking molds. The rising step was carried out in an LTE-8/00 60X40 oven (Salva Industrial, Guipúzcoa, Spain) at a relative humidity of 70 % and a temperature of 30 °C for 45 min. Finally, the dough was baked at 180 °C for 45 min in a LT-4/05 I 60X40 oven (Salva Industrial, Guipúzcoa, Spain). The resulting baked breads were cooled at room temperature for 1 h and then pre-frozen at -80 °C for 24 h in an ultra-freezer ULT40086 (Infrico medcare, Córdoba, Spain) before being subjected to freeze-drying in a LyoBench freeze-dryer (Noxair Life Sciences S.L., Barcelona, Spain) for 96 h at -50 °C and 0.076 mbar. After freeze-drying, the samples were ground using an A11 Basic analytical mill (IKA, Staufen, Germany) and sieved to a homogeneous particle size of 1 mm. The final product was stored in a desiccator at room temperature until the analysis.

All samples were analyzed in triplicate to assess the precision of the results and estimate their associated uncertainty.

Table S1. Retention time and mass spectrum parameters for the analysis of 29 alkaloids using the UHPLC-IT-MS/MS method described in this work.

Analyte	Retention Time (min)	Precursor Ion (*m/z*)	Isolation (width)	Reaction cut-off	Fragmentation Amplitude	MS^2 Predominant Product Ions (m/z)
Morphine	2.52	286	4.0	90	1.00	201; 183; 211; 229; 268; 155; 173
Intermedine	4.39	300	4.0	90	0.70	138; 120; 156; 256; 210; 282
Lycopsamine	4.75	300	4.0	90	0.70	138; 120; 156; 256; 210; 283
Europine	5.36	330	4.0	120	0.80	254; 138; 312; 156; 272
Europine *N*-oxide	5.92	346	4.0	120	0.80	328; 172; 270; 256; 138; 212
Codeine	6.01	300	4.0	95	0.95	215; 183; 225; 282; 243; 187; 199; 193; 164
Scopolamine	6.11	304	4.0	90	1.00	138; 156; 110; 274; 228; 94
Intermedine *N*-oxide	6.36	316	4.0	100	0.80	172; 226; 138; 155; 272; 298; 210
Lycopsamine *N*-oxide	6.52	316	4.0	100	0.80	172; 226; 138; 155; 272; 298; 112; 210; 254
Retrorsine	7.27	352	4.0	120	0.80	324; 276; 138; 304; 220; 151; 172
Oripavine	7.60	298	4.0	95	1.00	267; 249; 237; 223
Retrorsine *N*-oxide	7.76	368	4.0	130	0.90	340; 246; 220; 218; 202; 292; 238; 136; 178
Seneciphylline	7.87	334	4.0	110	0.80	306; 288; 120; 138; 151
Heliotrine	7.92	314	4.0	100	0.70	138; 120; 156; 297; 238; 193
Seneciphylline *N*-oxide	8.46	350	4.0	100	0.75	322; 118; 246; 137; 288; 228; 154; 274
Heliotrine *N*-oxide	8.47	330	4.0	120	1.00	172; 137; 298; 201; 265; 228
Atropine	8.94	290	4.0	85	0.95	124; 260; 93; 214; 91; 242
Senecivernine	9.15	336	4.0	110	0.80	308; 120; 138; 290; 153; 220; 248; 200
Senecionine	9.20	336	4.0	110	0.80	308; 120; 138; 290; 153; 220; 238
Senecivernine *N*-oxide	9.42	352	4.0	120	0.80	324; 220; 202; 136; 248; 218; 176
Senecionine *N*-oxide	9.78	352	4.0	120	0.81	324; 220; 246; 202; 136; 254; 152; 178 290
Thebaine	10.83	312	4.0	100	0.70	249; 281; 266; 221; 255; 237; 177; 126
Echimidine *N*-oxide	10.93	414	4.0	110	0.70	253; 395; 352; 338; 310
Echimidine	11.09	398	4.0	100	0.70	120; 220; 336; 380; 316

Analyte	Retention Time (min)	Precursor Ion (*m/z*)	Isolation (width)	Reaction cut-off	Fragmentation Amplitude	MS^2 Predominant Product Ions (m/z)
Senkirkine	11.49	366	4.0	130	0.80	<u>168</u>; 150; 338; 250; 186; 220; 268; 322
Noscapine	12.47	414	4.0	140	1.00	<u>220</u>; 336; 205; 353; 394
Lasiocarpine	12.59	412	4.0	100	0.70	<u>336</u>; 220; 120; 394; 238
Papaverine	13.00	340	4.0	110	1.00	<u>202</u>; 324; 171; 296; 147
Lasiocarpine *N*-oxide	13.07	428	4.0	100	0.80	<u>410</u>; 352; 254; 338; 302; 120; 278; 226

Ions underlined were used for quantification. Isolation width (*m/z*) is 4. Chromatographic conditions: 0-0.5 min 5% solvent B, 0.5-3 min 10% solvent B, 3-7 min 25% solvent B, 7-9 min 30% solvent B, 9-12 min 70% solvent B, 12-14 min 5% solvent B, and 14-15 min 5% solvent B. Mobile phase: 0.2% formic acid in water (*v/v*) (solvent A) and 0.2% ammonia in methanol (*v/v*) (solvent B). Flow rate 0.300 mL/min. Column temperature 30 °C. Total run time analysis 15 min.

Table S2. Instrumental validation parameters for UHPLC-IT-MS/MS analysis of tropane, pyrrolizidine and opium alkaloids.

Analytes	Linear range (µg/L)	Calibration line (R^2)	LOD (µg/L)	LOQ (µg/L)
Atropine	1.00 - 300	y= 286079x - 96474 (0.999)	0.10	0.33
Codeine	1.50 - 300	y= 112511x + 147551 (0.999)	0.33	1.10
Echimidine	1.00 - 300	y= 275410x + 67553 (0.999)	0.10	0.30
Echimidine *N*-oxide	1.00 - 300	y= 505743x - 189971(1)	0.07	0.22
Europine	1.00 - 300	y= 243156x + 3938 (0.999)	0.11	0.37
Europine *N*-oxide	1.00 - 300	y= 373611x + 33589 (0.999)	0.27	0.90
Heliotrine	0.30 - 300	y= 229561x + 123109 (0.999)	0.12	0.38
Heliotrine *N*-oxide	0.10 - 300	y= 278764x - 67688 (0.999)	0.03	0.10
Intermedine	1.00 - 300	y= 135755x + 106800 (0.999)	0.11	0.37
Intermedine *N*-oxide	1.00 - 300	y= 177412x + 170008 (0.999)	0.03	0.10
Lasiocarpine	1.00 - 300	y= 563983x + 493332 (0.999)	0.17	0.58
Lasiocarpine *N*-oxide	1.00 - 300	y= 624259x + 491699 (0.999)	0.11	0.37
Lycopsamine	1.50 - 300	y= 147211x + 10118 (0.999)	0.31	1.03
Lycopsamine *N*-oxide	1.00 - 300	y= 185438x + 236082 (0.999)	0.19	0.63
Morphine	1.00 - 300	y= 92204x + 22514 (0.999)	0.19	0.64
Noscapine	1.00 - 300	y= 6000000x - 8333333 (0.999)	0.06	0.18
Oripavine	1.50 - 300	y= 25663x + 127233 (0.999)	0.40	1.31
Papaverine	1.00 - 300	y= 897123x + 661681 (1)	0.05	0.15
Retrorsine	1.00 - 300	y= 162860x + 108780 (1)	0.08	0.26
Retrorsine *N*-oxide	1.00 - 300	y= 36593x + 142861 (0.999)	0.24	0.80
Scopolamine	1.00 - 300	y= 127690x + 151224 (0.999)	0.14	0.50
Senecionine	1.50 - 300	y= 374724x - 132869 (0.999)	0.38	1.28
Senecionine *N*-oxide	1.00 - 300	y= 58646x + 81583 (0.999)	0.16	0.54
Seneciphylline	1.00 - 300	y= 124404x + 293439 (0.999)	0.19	0.63
Seneciphylline *N*-oxide	1.00 - 300	y= 55456x + 85438 (0.999)	0.20	0.66
Senecivernine	1.00 - 300	y= 324675x + 346274 (0.999)	0.19	0.63
Senecivernine *N*-oxide	1.00 - 300	y= 68075x + 53911 (0.999)	0.14	0.48
Senkirkine	1.00 - 300	y= 148713x - 65905 (0.999)	0.17	0.58
Thebaine	1.50 - 300	y= 49226x + 42908 (0.999)	0.38	1.25

LOQ: limits of quantification; LOD: limits of detection determined as 3 and 10 times the signal/noise ratio, respectively. Analytes dissolved in a mixture of 0.2% formic acid in water/0.2% ammonia in methanol (95/5, *v/v*).

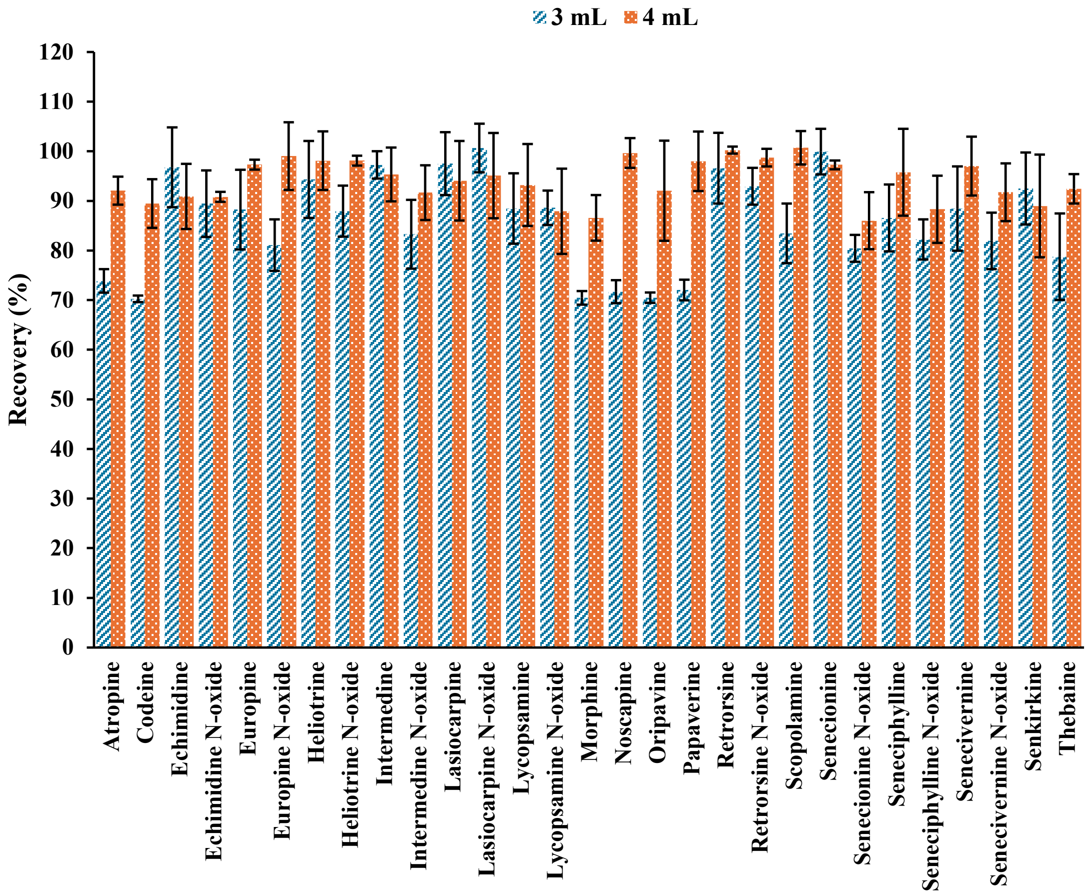

Figure S1. Recovery values (% ± SD) obtained from the solid-phase extraction (SPE) analysis of standard solutions (5 µg/kg tropane alkaloids, 400 µg/kg pyrrolizidine alkaloids and 1.5 mg/kg opium alkaloids) in acidified water (1% HCl) using 100 mg of RH-SBA-15-SO_3H-C18 as sorbent and different elution volumes (3 and 4 mL). SPE conditions: conditioning with 3 mL Milli-Q water, equilibration with 3 mL of 1% HCl in water, loading 5 mL of standard solution, vacuum drying for 3 min, elution with 3 or 4 mL of 5% ammonia solution in methanol, and vacuum drying for 3 min. Error bars represent the standard deviation of sample replicates (n=3).

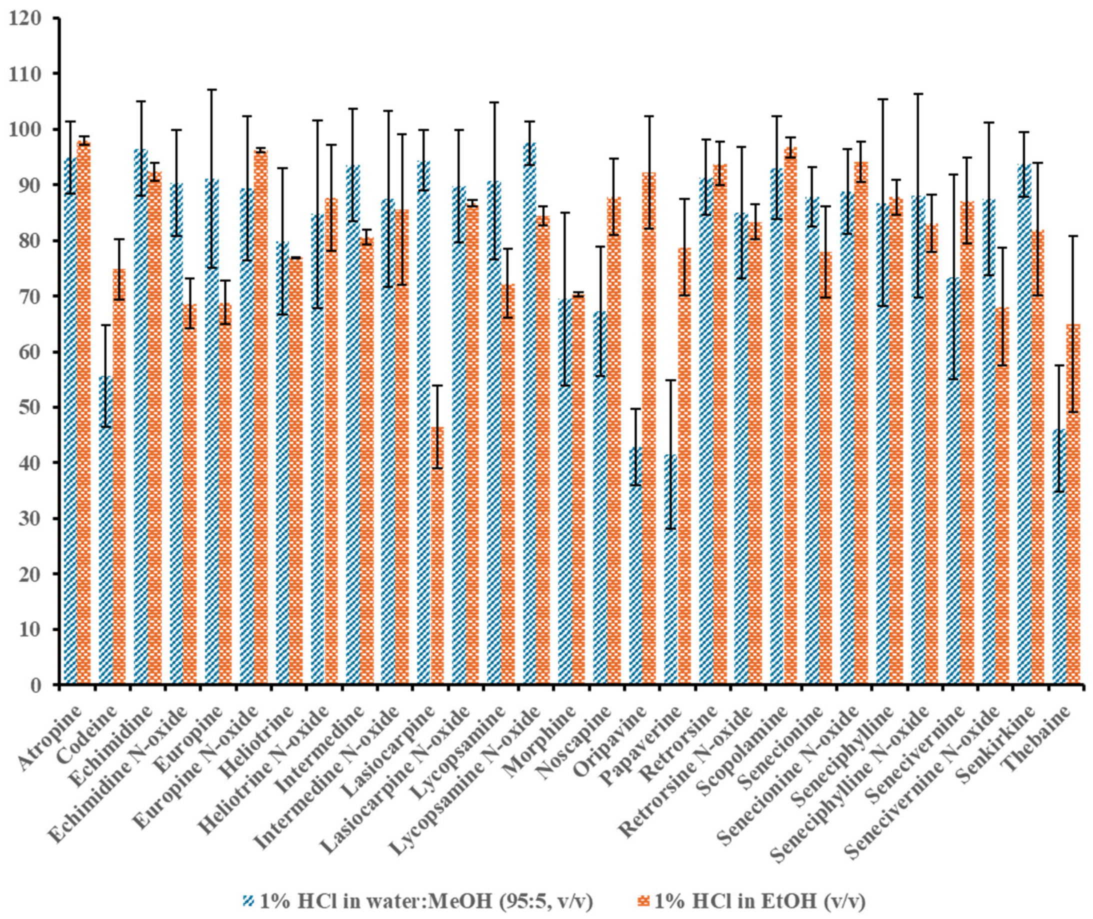

Figure S2. Recovery values (% ± SD) obtained for solid–liquid extraction (SLE) of tropane, pyrrolizidine and opium alkaloids from the gluten-free bread sample fortified with the target analytes at 5 µg/kg, 400 µg/kg s and 1.5 mg/kg, respectively, using **(a)** water/methanol (95:5 *v*/*v*) with 1% HCl and **(b)** in ethanol with 1% HCl as extraction solvents. SLE conditions: 0.5 g sample, 5 mL extraction solvent, 30 min magnetic stirring, 10 min centrifugation at 9000 rpm, filtration and injection into the UHPLC-IT-MS/MS system. Error bars represent the standard deviation of sample replicates (n = 3).

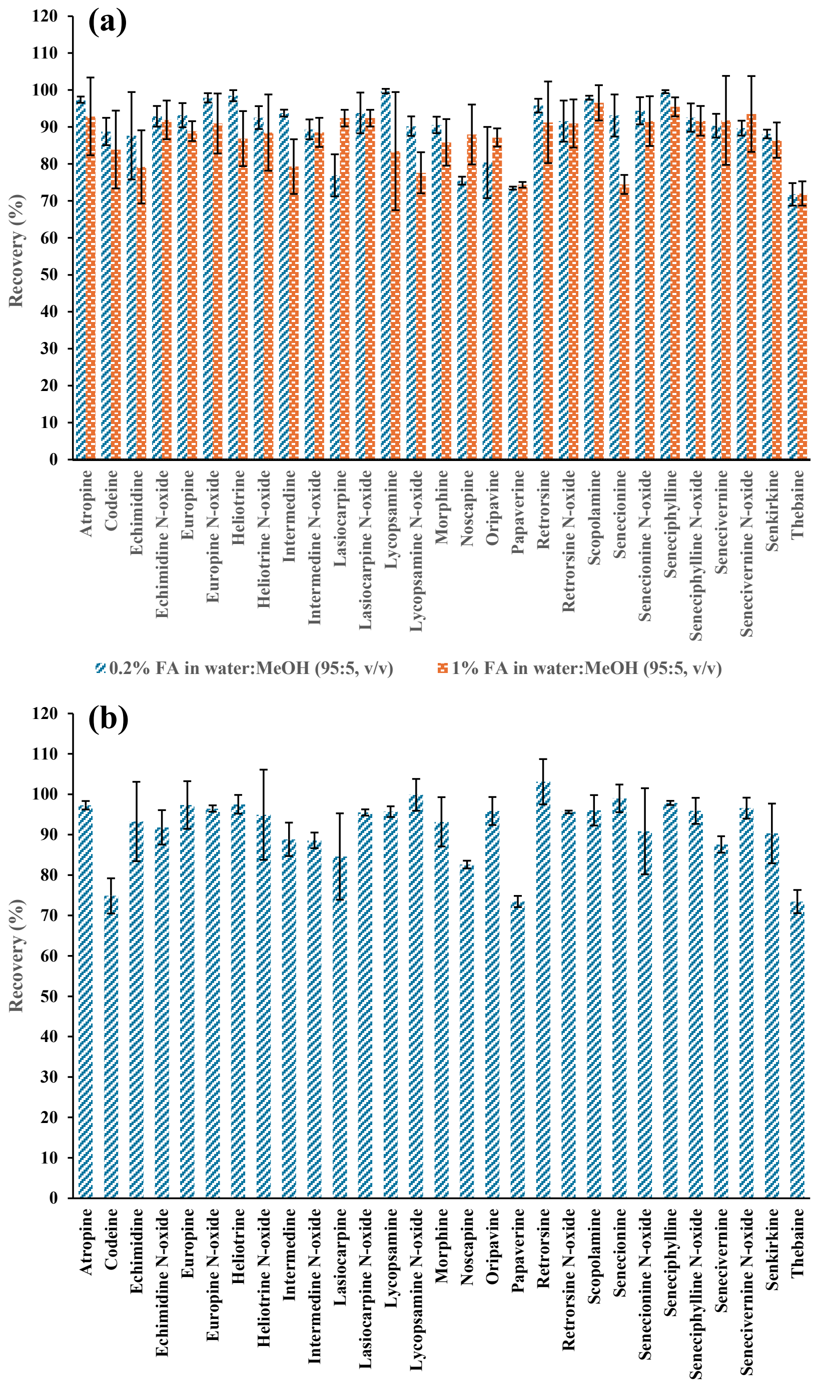

Figure S3. Recovery values (% ± SD) obtained for solid–liquid extraction (SLE) of tropane, pyrrolizidine and opium alkaloids from the gluten-free bread sample fortified with the target analytes at 5 µg/kg, 400 µg/kg and 1.5 mg/kg, respectively, using **(a)** water/methanol (95:5 *v*/*v*) with 0.2 and 1% formic acid and **(b)** water with 0.2% formic acid as extraction solvents. SLE conditions: 0.5 g sample, 5 mL extraction solvent, 30 min magnetic stirring, 10 min centrifugation at 9,000 rpm, filtration and injection into the UHPLC-IT-MS/MS system. Error bars represent the standard deviation of sample replicates (n = 3).

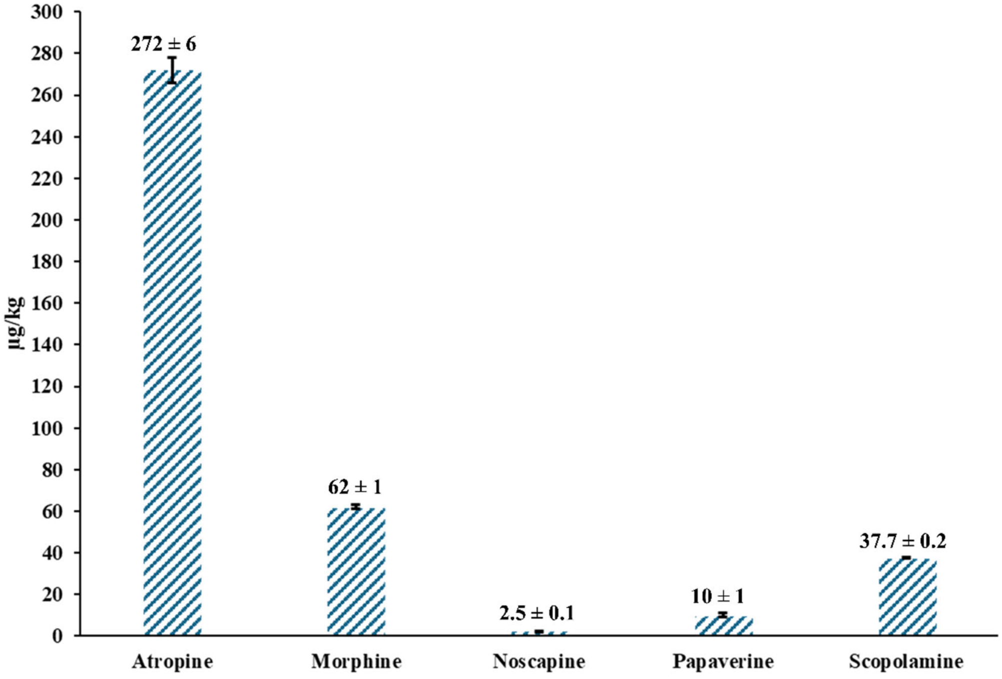

Figure S4. Total content (µg/kg) of tropane and opium alkaloids quantified in the gluten-free bread sample.

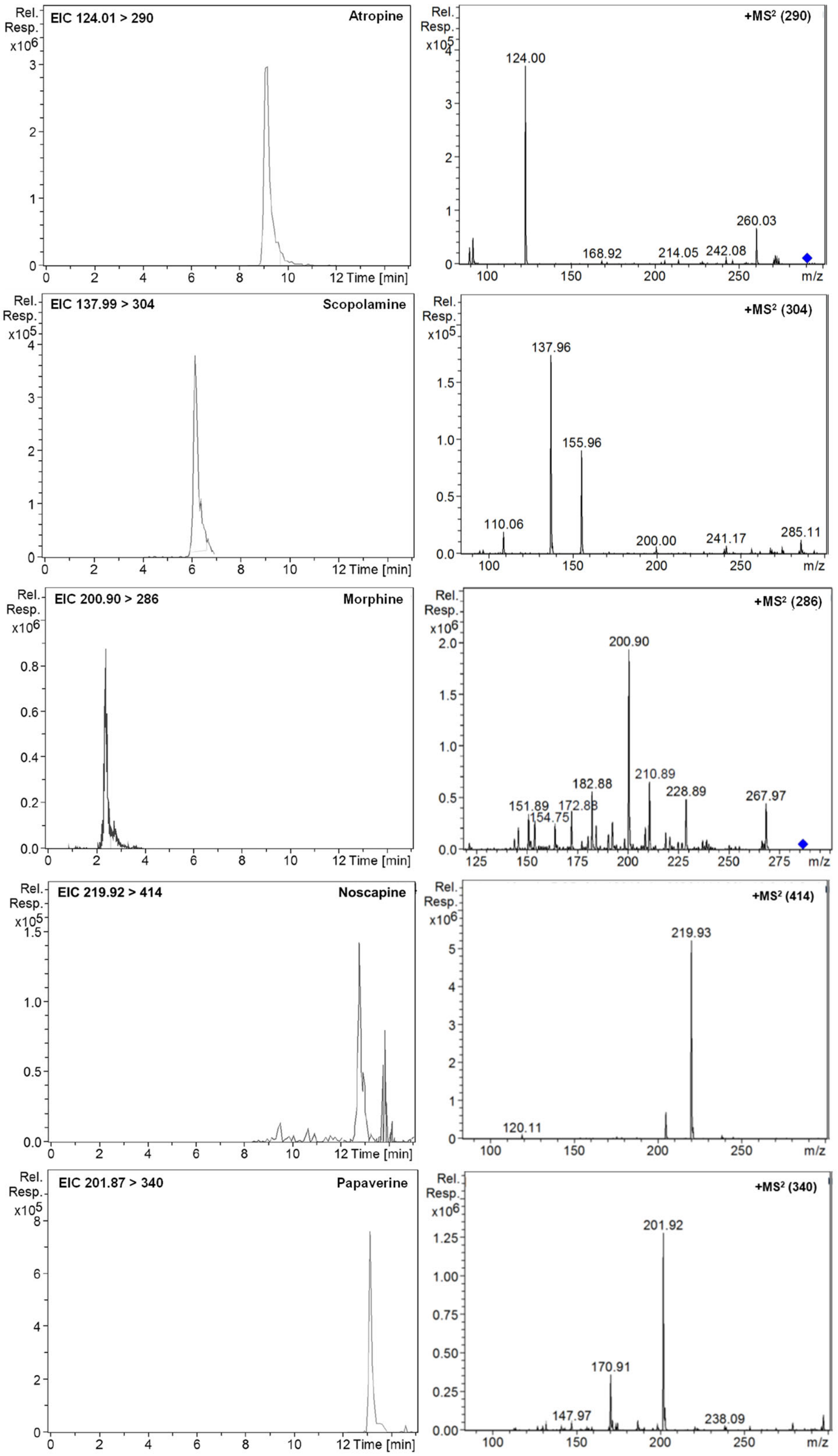

Figure S5. Extracted Ion Chromatograms (EICs) and mass spectra (MS^2) of atropine, scopolamine, morphine, noscapine, and papaverine in a standard solution. At the top of each EIC, the isolated precursor ion (*m/z*) is indicated on the left along with its extracted product ion to obtain the MS^2, while the name of the corresponding analyte is indicated on the right. At the top of each mass spectrum, the isolated precursor ion (*m/z*) is indicated on the right along with the ionization mode.
